# Spreading the Clinical Window for Diagnosing Fetal-Onset Hypogonadism in Boys

**DOI:** 10.3389/fendo.2014.00051

**Published:** 2014-05-07

**Authors:** Romina P. Grinspon, Nazareth Loreti, Débora Braslavsky, Clara Valeri, Helena Schteingart, María Gabriela Ballerini, Patricia Bedecarrás, Verónica Ambao, Silvia Gottlieb, María Gabriela Ropelato, Ignacio Bergadá, Stella M. Campo, Rodolfo A. Rey

**Affiliations:** ^1^Centro de Investigaciones Endocrinológicas “Dr. César Bergadá” (CEDIE), CONICET, FEI, División de Endocrinología, Hospital de Niños Ricardo Gutiérrez, Buenos Aires, Argentina

**Keywords:** hypopituitarism, cryptorchidism, micropenis, disorder of sex development, testosterone

## Abstract

In early fetal development, the testis secretes – independent of pituitary gonadotropins – androgens and anti-Müllerian hormone (AMH) that are essential for male sex differentiation. In the second half of fetal life, the hypothalamic–pituitary axis gains control of testicular hormone secretion. Follicle-stimulating hormone (FSH) controls Sertoli cell proliferation, responsible for testis volume increase and AMH and inhibin B secretion, whereas luteinizing hormone (LH) regulates Leydig cell androgen and INSL3 secretion, involved in the growth and trophism of male external genitalia and in testis descent. This differential regulation of testicular function between early and late fetal periods underlies the distinct clinical presentations of fetal-onset hypogonadism in the newborn male: primary hypogonadism results in ambiguous or female genitalia when early fetal-onset, whereas it becomes clinically undistinguishable from central hypogonadism when established later in fetal life. The assessment of the hypothalamic–pituitary–gonadal axis in male has classically relied on the measurement of gonadotropin and testosterone levels in serum. These hormone levels normally decline 3–6 months after birth, thus constraining the clinical evaluation window for diagnosing male hypogonadism. The advent of new markers of gonadal function has spread this clinical window beyond the first 6 months of life. In this review, we discuss the advantages and limitations of old and new markers used for the functional assessment of the hypothalamic–pituitary–testicular axis in boys suspected of fetal-onset hypogonadism.

The concept of male hypogonadism is usually associated with the adult patient, and rarely thought of as a condition in the prepubertal boy. Furthermore, male hypogonadism is most frequently equated to hypoandrogenism. Androgens are the dean of testicular hormones, and the normal testis produces very little or no testosterone during most of infancy and childhood. It is therefore easy to understand why the term hypogonadism is almost absent from the pediatrician’s terminology. However, many hypogonadal states in the male bear their origin in fetal life. With the advent of direct markers of Sertoli cell function, hypogonadism can be identified in boys beyond the early post-natal critical window of pituitary–gonadal activation (1) – called “mini-puberty” by some authors – and before pubertal age. In this review, we address the diagnostic approaches of fetal-onset male hypogonadism based on the physiology and pathophysiology of the hypothalamic–pituitary–testicular axis ontogeny.

## Ontogeny of the Hypothalamic–Pituitary–Testicular Axis

### Fetal life: The first versus the second and third trimesters

The gonadotropin-releasing hormone (GnRH) neurons derive from cells present in the nasal placode in the sixth fetal week (2), which migrate together with olfactory axons and blood vessels through the cribriform plate and arrive in the developing forebrain in the 9th–10th weeks. Several genes are involved in the development and migration of GnRH neurons, including *KAL1*, *FGF8*, *FGFR1*, *PROK2*, *PROKR2*, *CHD7*, *WDR11*, and *NELF*, and in their homeostasis and function, including *DAX1* (or *NR0B1*), *LEP*, *LEPR*, *KISS1*, *KISS1R*, *TAC3*, *TACR3*, and *GNRH1* [reviewed in Ref. (3, 4)].

The pituitary gonadotropes develop in the Rathke’s pouch following a sequential differentiating pathway, which also includes the other pituitary cell lineages, from the oral ectoderm ancestor. Early genes, like *SHH*, *GLI1*, *GLI2*, *LHX3*, *LHX4*, *PITX1*, *PITX2*, *OTX2*, and *HESX1*, are involved in the differentiation of all pituitary cell lineages, whereas *TBX19* (or *TPIT*), *GATA2*, and *SF1* (or *NR5A1*) are more specifically related to the gonadotrope lineage [reviewed in Ref. (5)]. Fully functional gonadotropes are present in the fetal male pituitary and secrete luteinizing hormone (LH) from week 12 and Follicle-stimulating hormone (FSH) from week 14 (6). Circulating levels of both gonadotropins increase to attain peak levels by weeks 20–25 and then decrease toward term (7–9).

The testes differentiate from the adreno-gonadal primordium by the seventh week of gestation. Interestingly, Sertoli cells actively secrete anti-Müllerian hormone (AMH), involved in the regression of the uterine anlage during the eighth and ninth weeks, i.e., before exposure to FSH. In fact, basal AMH expression is triggered by SOX9 and enhanced by SF1, GATA4, and WT1 independent of FSH [reviewed in Ref. (10)]. Afterward, FSH increases testicular AMH output by inducing Sertoli cell proliferation and AMH transcription following the classic FSH receptor transduction pathway involving protein kinase A and cyclic AMP (10, 11). Sertoli cells also secrete inhibin B, which is present at high levels in the serum of mid-term fetuses and only slightly lower by term (8, 9). Sertoli cells are not directly regulated by androgens during fetal life since they do not express the androgen receptor [reviewed in Ref. (12)].

**Figure 1:**
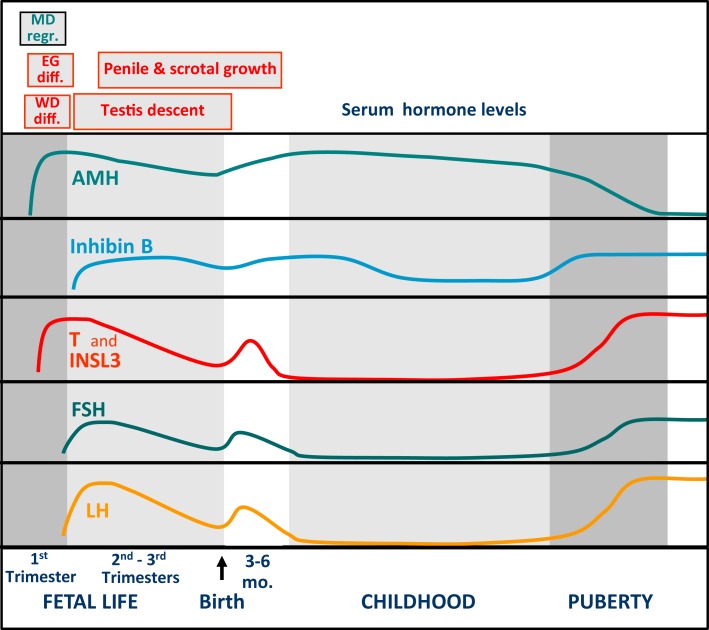
**Schematic representation of the pituitary–testicular axis hormone levels and of sexual differentiation and development of male internal and external genitalia**. WD diff., Wolffian duct differentiation; MD regr., Müllerian duct regression; EG diff., differentiation of the external genitalia.

Approximately 1 week later than Sertoli cells do, Leydig cells differentiate in the interstitial tissue and secrete testosterone, responsible for the differentiation of the male gonaduct, the prostate, and the external genitalia, independently from fetal pituitary LH. In fact, the major regulator of testosterone production during the first trimester is chorionic gonadotropin (hCG), which circulates at high levels in fetuses with a peak at 12–17 weeks subsequently decreasing through term (7, 13). The relevance of fetal LH in Leydig cell function becomes more evident during the second and third trimesters. Both LH and hCG act on the same transmembrane receptor, the LHCG-R, present on the Leydig cell membrane and inducing cell proliferation and differentiation as well as androgen and insulin-like 3 (INSL3) synthesis and secretion. Male differentiation of internal and external genitalia is completed in the first trimester (Figure 1). Afterward, androgens induce the growth of the phallus and the trophism of the scrotum, whereas both androgens and INSL3 are important for testicular descent (14).

### Post-natal life: Infancy, childhood, and puberty

**Figure 2:**
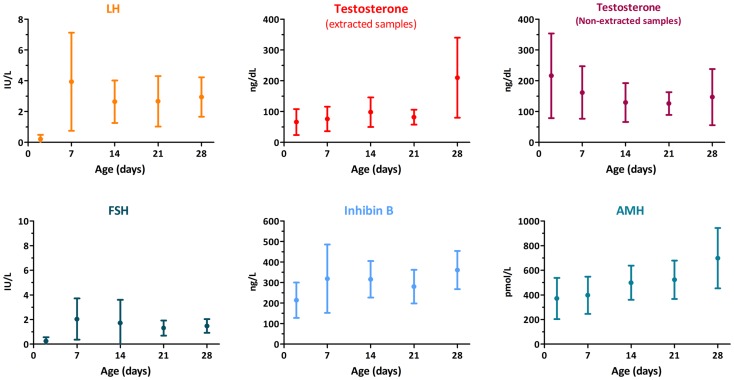
**Serum levels of gonadotropins and testicular hormones in male newborns**. Data obtained from Ref. (15).

The decreasing trend in the whole hypothalamic–pituitary–testicular axis activity is reflected in low perinatal levels of all hormones (Figure 2) (9). Thereafter, an increase in circulating levels is observed in the neonate already by the end of the first week for gonadotropins, and from the second to fourth weeks for AMH, inhibin B, and testosterone (15, 16). It should be noted for testosterone that serum samples must be extracted to avoid interferences that artificially overestimate results (Figure 2). LH drives testosterone and INSL3 to peak levels during the third month; thereafter, they all decline and attain very low or undetectable levels after the sixth month (Figure 1) (16–18). Assays for INSL3 are now commercially available, with sufficient sensitivity to be used in patients during childhood (19), although an hCG test may be needed.

On the other hand, FSH continues to induce Sertoli cell proliferation resulting in a continuous increase in testis volume. Androgens may also exert a proliferative effect on Sertoli cells (20), but the effect should be indirect since the androgen receptor is still not expressed in Sertoli cells during early infancy [reviewed in Ref. (12)]. It should be noted that the absolute volume increment described in this period of life is modest ( <1.5 mL) and cannot be clinically evidenced by palpation (21). AMH and inhibin B secretion is also enhanced: the levels of both hormones increase progressively through infancy (Figure 1) (15, 22, 23). The increase observed during the first months of life may be linked to the marked proliferation of Sertoli cells that occurs after mid-gestation with a further increment after birth (24), probably enhanced by the post-natal gonadotrophic surge. Serum inhibin B levels are as high as those observed in pubertal boys during the first 6 months of age; thereafter, a progressive fall occurs until the age of 4–6 years, but serum concentrations remain considerable, since they are above the lowest limit of normal adult range (23), and can be readily detected with the commercially available new generation assays (25). Serum AMH peaks during the second year and then remains fairly stable during childhood (22, 26). Altogether, these data clearly indicate that Sertoli cells are functionally active during infancy and childhood.

**Figure 3:**
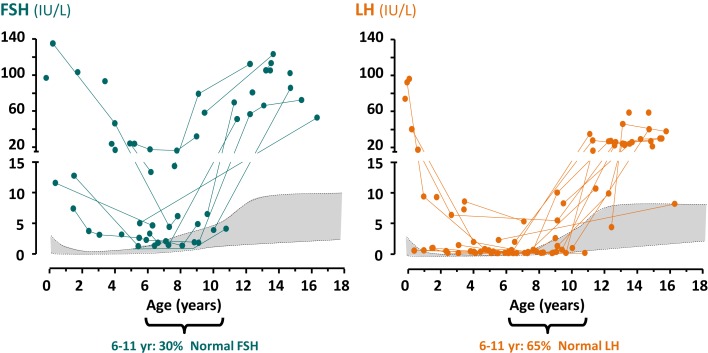
**Serum levels of gonadotropins in anorchid boys**. Reproduced from Ref. (29), ©2012 Blackwell Publishing Ltd., with permission from Blackwell Publishing Ltd., John Wiley and Sons.

Testosterone and inhibin B are the most relevant physiological factors involved in gonadotropin negative feedback in the adult. A possible role for inhibin B in FSH negative feedback before puberty is still a matter of debate. Higher FSH than LH levels observed in boys with no functional gonadal tissue (27–29), the inverse correlation between FSH and inhibin B levels observed in cryptorchid boys (30), and the suppression of FSH secretion observed in prepubertal patients with Sertoli cell neoplastic proliferations and increased inhibin B (31) support the hypothesis of the active role that inhibin B has in regulating FSH. However, the decrease in LH and FSH levels during normal male childhood is not fully dependent on these testicular hormones, since it also occurs in a considerable proportion of boys with gonadal dysgenesis (27) or anorchia (Figure 3) (29).

A progressive increase in gonadotropin pulse amplitude and frequency occurring between 9 and 14 years of age triggers testicular pubertal maturation. LH induces Leydig cells androgen production again: intratesticular testosterone concentration increases and acts on Sertoli cells, which now express the androgen receptor. Consequently, they acquire a mature phenotype characterized by the development of the blood–testis barrier and a down-regulation of AMH production [reviewed in Ref. (12)]. The rise in serum testosterone occurs 1–2 years later (32, 33). Germ cells, hitherto limited to spermatogonia, enter meiosis and go through the complete spermatogenic process giving rise to spermatozoa. Spermatogenic development is responsible for the remarkable increase of testis volume during puberty. FSH and germ cells induce an increase in inhibin B. Serum levels of inhibin B increase concomitantly with testicular volume, and attains adult levels as early as pubertal stage II (23, 34, 35). INSL3 secretion also increases during puberty (36); in adult, the production and secretion of INSL3 is maintained by the long-term trophic effect of LH on Leydig cell structure and function and independent of the acute steroidogenic effect of LH (16).

## Definition and Classification of Congenital Male Hypogonadism

From the comprehension of the changes occurring in the normal physiology of the pituitary–testicular axis during pre- and post-natal life, it emerges clearly that male hypogonadism cannot be limited to hypoandrogenism. The definition should be extended to all situations characterized by a decreased testicular function, as compared to what is expected for age, involving an impaired hormone secretion by Leydig cells (androgens, INSL3) and/or Sertoli cells (AMH, inhibin B) and/or a disorder of spermatogenesis (Table 1).

**Table 1:** **Classification of fetal-onset male hypogonadism**.

	Whole gonadal dysfunction	Cell-specific gonadal dysfunction
**PRIMARY HYPOGONADISM**		
First trimester	Gonadal dysgenesis	Leydig cells
		LHCG-R mutation
		Steroidogenic protein defects
		Sertoli cells
		AMH mutation
Second – third trimesters	Testicular regression syndrome	Leydig cells INSL3 mutation
	Testicular torsion	
	Endocrine disruptors	Sertoli cells
		FSH-R mutation
**CENTRAL HYPOGONADISM**		
Second – third trimesters	Multiple pituitary hormone deficiency	Leydig cells LHβ-subunit gene mutation Neurokinin defects
	Isolated hypogonadotropic hypogonadism (IHH)	
		Sertoli cells
		FSHβ-subunit gene mutation
**COMBINED HYPOGONADISM**		
First trimester	DAX1 gene mutations	None
Second – third trimesters	Prader–Willi syndrome	None

It should also be considered that the clinical manifestations of male hypogonadism will vary according to: (a) the level of the hypothalamic–pituitary–testicular axis primarily affected, (b) the testicular cell population initially impaired, and (c) the period of life when the condition is established (37).

### Level of the axis primarily affected: Central, primary, or combined hypogonadism

In central (or hypothalamic–pituitary) hypogonadism, testicular failure is secondary to a disorder affecting the secretion of GnRH or gonadotropins. It is usually characterized by an impaired production of both LH and FSH, and thus called hypogonadotropic hypogonadism; however, as discussed later, this nomenclature is not always applicable, and some cases of central hypogonadism may present with normal or even increased levels of one gonadotropin.

In primary hypogonadism, the testis is the primarily affected organ. This may lead to an impaired production of testicular hormones and a disruption of the negative feedback to the hypothalamic–gonadotrope axis, which results in an elevation of FSH and/or LH. In adult endocrinology, primary hypogonadism is usually identified as hypergonadotropic; however, as discussed in the previous section, during childhood, primary hypogonadism – or even agonadism – may present with normal gonadotropin levels (27–29).

In certain disorders, both the hypothalamic–gonadotrope axis and the testis are affected concomitantly, e.g., in *DAX1* mutations or in oncologic patients exposed to cranial radiotherapy and chemotherapy. These “dual” conditions are characterized by a lack of gonadotropin elevation during puberty or adulthood in spite of the low testicular secretion of androgens and/or inhibin B [reviewed in Ref. (37)].

### Whole testicular versus spermatogenic, Leydig cell-specific or Sertoli cell-specific failure

Whole testicular failure or hypogonadism reflects the concomitant impairment of all testicular cell populations. On the contrary, the disorder may primarily involve only one testicular cell population; for instance, spermatogenic-specific failure results from Yq chromosome deletions, steroidogenic failure from defects in LH, its receptor or steroidogenic enzymes, and Sertoli cell-specific hypogonadism from defects in FSH or its receptor or in the AMH gene, as we discuss more in detail below.

### Onset of male hypogonadism: Fetal versus post-natal life

Male hypogonadism can be congenital, i.e., fetal-onset hypogonadism, or result from a condition acquired during post-natal life. The clinical presentation depends on the period of life in which testicular failure is established. In adulthood, androgen deficiency leads to decreased libido, impotence, fatigue, loss of bone and muscle mass, increased fat mass and metabolic disorders, and spermatogenic failure results in oligo- or azoospermia. At pubertal age, male hypogonadism results in the absence or the arrest of pubertal development. Because the hypothalamic–pituitary–steroidogenic function is normally low during childhood – as explained above – male hypogonadism remains clinically unapparent when established in this period of life unless suspected and actively sought for by measuring serum AMH or inhibin B in basal conditions, or testosterone or INSL3 after stimulation with hCG [reviewed in Ref. (37)]. Fetal-onset hypogonadism may lead to a variety of clinical presentations, which are discussed in detail below.

## Pathophysiology of Fetal-Onset Male Hypogonadism

The clinical consequences of fetal-onset male hypogonadism can be deduced from the understanding of the normal ontogeny of the male reproductive axis during fetal life described above. When established in the first trimester, the lack or insufficient levels of testis hormones during the critical window of male sex differentiation (weeks 8–13) lead to disorders of sex development (DSD) presenting with female or ambiguous genitalia. Because Leydig cell androgen production is essentially under placental hCG – not fetal LH – control in the first trimester, central hypogonadism does not result in DSD. Primary hypogonadism established in the second half of gestation and central hypogonadism lead to a decreased number of Sertoli cells and also to an impaired testicular output of androgens and INSL3. The clinical consequences are microorchidism, micropenis, and cryptorchidism.

### Primary hypogonadism established in the first trimester

#### Whole testicular dysfunction: gonadal dysgenesis

Gonadal dysgenesis may result from chromosomal aberrations or mutations affecting genes controlling testicular differentiation (Table 2). Chromosome aberrations involving the short arm of the Y chromosome cause gonadal dysgenesis affecting all cell populations. Similarly, deletions of the short arm of chromosome 9 – where *DMRT1* and *DMRT2* map (38) – and duplications of Xp21.3-p21.2 – where *DAX1* gene maps (39) and of 1p31-p35 – where *WNT4* maps (40) – result in testicular dysgenesis. 46,XY patients with mutations in *SRY* (41) or *MAMLD1* (42) also present with gonadal dysgenesis. Mutations in other genes associate testicular dysgenesis with dysfunctions of other organs (Table 2). *SF1* mutations may associate gonadal dysgenesis with adrenal failure (43), yet isolated testicular dysfunction can be observed (44). Mutations in *WT1* result in gonadal dysgenesis associated with degenerative renal disease, resulting in Denys–Drash syndrome or in Frasier syndrome (45). Haploinsufficiency of *SOX9* leads to a polymalformative syndrome including gonadal dysgenesis, bowing and angulation of long bones (known as campomelic dysplasia), hip dislocation, hypoplastic scapula, small thoracic cage, macrocephaly, facial dysmorphism, and cardiac and renal defects (40, 46). Homozygous mutations of *DHH* gene result in the association of gonadal dysgenesis and minifascicular neuropathy (47, 48). Mutations in *XH2* gene cause the ATRX syndrome, characterized by α-thalassemia, mental retardation, facial dysmorphism and gonadal dysgenesis (49). Recently, *MAP3K1* mutations have been identified as another cause of partial or complete gonadal dysgenesis (50). Finally, mutations in *TSPYL1* have been found in patients with gonadal dysgenesis and sudden death (51). However, the vast majority of dysgenetic DSD cases remain unexplained, which suggests that several other gene defects may be the underlying cause.

**Table 2:** **Clinical features in male patients with fetal-onset primary hypogonadism with whole gonadal dysfunction**.

Affected chromosome	Gene	OMIM	Associated clinical features
9p24 deletion	*DMRT1* and *DMRT2*	#154230	Dysgenetic DSD
			Mental retardation, microcephaly, facial malformations, short stature
			Digestive or bronchial malformations
Xp21 duplication	*DAX1* = *NR0B1* and other genes	#300018	Dysgenetic DSD
1p31-p35 duplication	*WNT4* and other genes	*603490	Dysgenetic DSD
Yp11.31	*SRY*	*48000	Dysgenetic DSD
Xq28	*MAMLD1*	*300120	Dysgenetic DSD
9q33.3	*SF1* = *NR5A1*	+184757	Dysgenetic DSD
			Adrenal insufficiency
11p13	*WT1*	#136680	Dysgenetic DSD
		#194072	Renal dysgenesis/tumor (Denys–Drash, Frasier and WAGR syndromes)
		#194080	
17q24.3	*SOX9*	#114290	Dysgenetic DSD
			Campomelic dysplasia
12q13.12	*DHH*	#233420	Dysgenetic DSD
			Minifascicular neuropathy
Xq21.1	*ATRX* = *XH2*	#301040	Dysgenetic DSD
			Mental retardation, α-thalassemia
5q11.2	*MAP3K1*	#613762	Dysgenetic DSD
6q22.1	*TSPYL1*	#608800	Dysgenetic DSD
			Sudden infant death

*DSD, disorder of sex development; OMIM, Online Mendelian Inheritance in Man locus, gene and phenotype numbers (http://www.ncbi.nlm.nih.gov/omim)*.

Exposure to environmental disruptors *in utero* has also been implicated as the underlying cause for interlinked reproductive disorders like cryptorchidism, hypospadias, infertility and testicular cancer, which seem to show an increasing trend. This association is known as the testicular dysgenesis syndrome (52).

When the gonadal dysgenesis is complete, internal and external genitalia differentiate along the female pathway since the streak gonads do not secrete any androgens or AMH. These 46,XY girls are apparently normal and do not seek medical attention until pubertal age when they present with absence of telarche and menarche. Only in the case of contradiction between a karyotype performed during gestation and the lack of virilization, does the case present to the specialist immediately after birth.

In partial forms of testicular dysgenesis, the degree of undervirilization depends on the amount of functional gonadal tissue the patient has. The external genitalia may be more or less ambiguous, testes do not descend and Wolffian derivatives are more or less atrophic as signs of insufficient androgen secretion, reflecting Leydig cell dysfunction. The persistence of müllerian derivatives reflects defective AMH production as a sign of Sertoli cell dysfunction.

In both complete and partial forms, the androgen and inhibin B feedback mechanisms are insufficient and the gonadotrope secretion of gonadotropin is exaggerated.

#### Leydig cell-specific dysfunction: isolated fetal hypoandrogenism

When only Leydig cell development and/or function are primarily disturbed in the first trimester of fetal life, insufficient androgen production results in undervirilisation and cryptorchidism. On the contrary, Sertoli cells are normally active and secrete AMH which induces full regression of Müllerian ducts. Therefore, this apparently normal girl has no uterus and a short blind-end vagina. Similar to complete gonadal dysgenesis, these patients seek medical attention at pubertal age because of the absence of telarche and primary amenorrhea. In the cases of a partial defect, androgen secretion is insufficient to virilize the fetus adequately: the newborn has ambiguous external genitalia and hypotrophic Wolffian duct derivatives. The degree of virilization is commensurate with the residual steroidogenic activity of the gonads. The gonadotrope secretes excessive gonadotropins with an increased LH:FSH ratio, because FSH is negatively regulated by inhibin B.

Leydig cell aplasia is a rare form of isolated fetal hypoandrogenism leading to a DSD due to inactivating mutations of the LHCG-R (Table 3) [reviewed in Ref. (53)]. Defective androgen production by the testis can also result from mutations in one of the five enzymatic activities necessary for the synthesis of testosterone from cholesterol (Table 3). Three of these are common to adrenal and gonadal steroidogenesis: cholesterol side-chain cleavage (P450scc), 3β-hydroxysteroid dehydrogenase (3β-HSD), and 17α-hydroxylase (P450c17). A deficiency in any of these in 46,XY individuals results in testicular hypoandrogenism leading to genital ambiguity and adrenal insufficiency leading to congenital adrenal hyperplasia. Two steroidogenic steps – 17,20-lyase (activity contained in P450c17) and 17β-hydroxysteroid dehydrogenase (17β-HSD) – are required only for gonadal steroidogenesis; therefore, their defects result only in hypovirilization without adrenal insufficiency [reviewed in Ref. (53)].

**Table 3:** **Clinical features in male patients with fetal-onset primary hypogonadism with Leydig cell-specific (steroidogenic) dysfunction**.

Gene	Protein	OMIM	Hormone levels	Associated clinical features
*LHCG-R*	LH/CG receptor	#238320	↓ ↓All steroids	None
*STAR*	StAR	#201710	↓ ↓All steroids	Lipoid congenital adrenal hyperplasia
*CYP11A1*	P450scc	#613743	↓ ↓All steroids	Adrenal insufficiency
*CYP17A1*	P450c17 (17α-hydroxylase activity)	#202110	↑Pregnenolone	Adrenal insufficiency
			↑Progesterone	Hypertension
*CYP17A1*	P450c17 (17,20-lyase activity)	#202110	↑17OH-pregnenolone	Adrenal insufficiency
			↑17OH-progesterone	
			↑Pregnenolone	
			↑Progesterone	
*POR*	P450 oxidoreductase	#613571	↑Progesterone	Antley–Bixler syndrome
			↑17OH-progesterone	
*HSD3B2*	3β-HSD type 2	#201810	↑DHEA	Adrenal insufficiency
			↑17OH-pregnenolone	
			↑Pregnenolone	
*HSD17B3*	17β-HSD type 3	#264300	↑Androstenedione	None
			↑DHEA	
			↑17OH-progesterone	
			↑17OH-pregnenolone	

*OMIM, Online Mendelian Inheritance in Man locus, gene and phenotype numbers (http://www.ncbi.nlm.nih.gov/omim)*.

#### Sertoli cell-specific dysfunction: AMH deficiency

The persistent Müllerian duct syndrome (PMDS) is a rare form of DSD characterized by persistence of Müllerian derivatives in otherwise normally virilized 46,XY individuals. Regression of Müllerian ducts normally occurs between 8 and 10 weeks of fetal development, under the influence of AMH produced by fetal Sertoli cells. If active AMH is not produced, owing to *AMH* gene mutations, Müllerian ducts develop into uterus, fallopian tubes, and upper vagina notwithstanding normal virilization of external genitalia and urogenital sinus. PMDS can also be consecutive to mutations of the AMH receptor type II gene (*AMHR2*), but in this case testicular function is normal [reviewed in Ref. (54)]. PMDS should not be considered in patients with defects in the virilization of external genitalia. Gonadotrope activity is not affected during fetal life.

### Primary hypogonadism established in the second and third trimesters

#### Whole testicular dysfunction: testicular regression syndrome

The existence of fully virilized external genitalia, i.e., completely fused scrotum and a urethral opening at the tip of the penis, is indicative of the existence of functional testes in the first trimester of gestation. However, the gonads may undergo regression (vanishing testes) due to torsion of the spermatic cord or to other unknown situations, resulting in a deficient or completely absent exposure to testicular hormones until the end of fetal life. The hypoandrogenism leads to scrotal hypotrophy and micropenis. Androgen and inhibin B insufficiency results in an exaggerated gonadotrope activity.

#### Leydig cell-specific dysfunction: INSL3 deficiency

Mutations in *INSL3* lead to a rare form of Leydig cell-specific dysfunction without hypoandrogenism. Newborns are normally virilized but present with cryptorchidism, reflecting the defect in testicular descent [reviewed in Ref. (55)]. Because INSL3 has no effect on the gonadotrope, LH and FSH secretion are not disturbed in these individuals during fetal life.

#### Sertoli cell-specific dysfunction: FSH receptor mutations

As already discussed, Sertoli cell differentiation in early fetal life is not dependent on FSH; therefore, male fetuses with FSH receptor mutations secrete sufficient amounts of AMH to induce Müllerian duct regression. On the contrary, since FSH is an important Sertoli cell mitogen, FSH-R mutations lead to Sertoli cell hypoplasia and small testes. Adults have low sperm count, low inhibin B, and moderately elevated FSH (56).

### Central hypogonadism established in the second and third trimesters

#### Whole testicular dysfunction: hypogonadotropic hypogonadism

As already discussed, deficient LH and FSH production by the fetal pituitary has no effect on sexual differentiation occurring in the ninth to thirteenth weeks of gestation, but do impact on genital development dependent on testicular function in the second and third trimesters of fetal life. Gonadotropin deficiency may result from an impaired differentiation of the gonadotrope in the context of a defective development of the pituitary primordium, and is therefore associated with multiple pituitary hormone deficiency. Alternatively, the defect may be restricted to the gonadotrope axis as a consequence of an impaired development, migration or function of the GnRH neurons, or of an impaired function of the gonadotrope. The lack of gonadotropin stimulus in this period of fetal development may result in small testes due to FSH deficiency, micropenis reflecting hypoandrogenism due to LH deficiency, and cryptorchidism as a sign of androgen and INSL3 insufficiency secondary to LH deficiency.

##### Multiple pituitary hormone deficiency

Congenital hypopituitarism occurs in approximately 1:4,000–1:10,000 newborns, with a 7:3 male-to-female ratio (57), and involves multiple pituitary cell lineages in approximately 80% of the cases. Mutations in genes involved in early pituitary differentiation and development usually result in multiple pituitary hormone deficiency including hypogonadotropic hypogonadism, usually due to pituitary hypoplasia. Although variable, there are a few clinical signs that may help in the identification of the underlying cause (Table 4) [reviewed in Ref. (58, 59)]. For instance, the association of congenital multiple pituitary hormone deficiency with septo-optic dysplasia (midline neural defects and optic nerve hypoplasia) has been observed in patients with mutations in *HESX1*, *SOX2* and *SOX3*. Midline defects, coloboma and polydactyly are also present in *HESX1* patients, anophthalmia or microphthalmia and esophageal atresia in *SOX2* cases, and X-linked mental retardation in *SOX3* mutations. *LHX3* defects are present in patients with rigid and short cervical spine; *LHX4* mutations can be found in individuals with abnormalities in the central skull base; *GLI2* in patients with holoprosencephaly; *PITX2* in patients with Axenfeld–Rieger syndrome (anomalies of anterior eye chamber, dental hypoplasia, craniofacial dysmorphism, and protuberant umbilicus); *SIX6* in patients with absent optic chiasm and brain cortical atrophy, and *OTX2* in patients with microphthalmia. Defects in late development factors, like *PROP1* are present in non-syndromic patients with multiple pituitary hormone deficiencies. Currently, only <15% of the etiologies of congenital hypopituitarism have been identified (60).

**Table 4:** **Clinical features in male patients with fetal-onset central hypogonadism associated with multiple pituitary hormone deficiency**.

Gene	OMIM	Other pituitary lineages affected	Associated clinical features
*HESX1*	#182230	Somatotrope	Septo-optic dysplasia
		Lactotrope	Midline defects
		Thyrotrope	Coloboma
		Corticotrope	Polydactyly
*SOX2*	#206900	Somatotrope	Septo-optic dysplasia
			Anopthalmia/microphthalmia
			Sensorineural defects
			Esophageal atresia
*SOX3*	#312000	Somatotrope	Septo-optic dysplasia
		Thyrotrope	
		Corticotrope	
*LHX3*	#221750	Somatotrope	Rigid and short cervical spine
		Lactotrope	Limited head rotation
		Thyrotrope	
*LHX4*	#262700	Somatotrope	Hindbrain defects
		Thyrotrope	Abnormality of central skull base
		Corticotrope	
*GLI2*	#610829	Somatotrope	Holoprosencephaly
		Lactotrope	
		Thyrotrope	
		Corticotrope	
*PITX2*	#180500	Somatotrope	Axenfeld–Rieger syndrome (anomalies of anterior eye chamber, dental hypoplasia, craniofacial dysmorphism and protuberant umbilicus)
		Thyrotrope	
*SIX6*	#212550	Somatotrope	Anophthalmia
			Brain cortical atrophy
			Brachiootorenal syndrome
			Oculoauriculovertebral spectrum
*OTX2*	#613986	Somatotrope	Microphthalmia/anophthalmia
		Thyrotrope	Cleft palate
		Corticotrope	Developmental delay
*PROP1*	#262600	Somatotrope	Intra- and extra-sellar cell mass, which may degenerate leading to empty sella later in life
		Thyrotrope	
		Corticotrope	

*OMIM, Online Mendelian Inheritance in Man locus, gene and phenotype numbers (http://www.ncbi.nlm.nih.gov/omim)*.

##### Isolated hypogonadotropic hypogonadism

Congenital isolated central hypogonadism can present as the only manifestation of the disorder (normosmic hypogonadotropic hypogonadism), or be associated with partial or complete loss of olfaction (Kallmann syndrome or anosmic hypogonadotropic hypogonadism), usually associated with other anatomical and/or neurological defects [reviewed in Ref. (61)].

Hyposmic/anosmic hypogonadotropic hypogonadism with or without other syndromic features results from mutations in the genes involved in the development and migration of the GnRH neurons from the olfactory placode to the hypothalamus [reviewed in Ref. (61)]. The insufficient GnRH production is associated with olfactory bulb hypoplasia or aplasia in magnetic resonance imaging. Associated clinical manifestations may change according to the defective gene: *KAL1*, *FGF8* and its receptor *FGFR1*, *PROK2* and its receptor *PROKR2*, *CHD7*, *NELF*, *HS6ST1*, *WDR11*, *SEMA3A* (Table 5) [reviewed in Ref. (4)].

**Table 5:** **Associated clinical features in male patients with fetal-onset isolated central hypogonadism due to defects in the migration of the GnRH neuron**.

Gene	OMIM	Associated clinical features
*KAL1*	#308700	Bimanual synkinesia, unilateral renal agenesis
		Less frequently: palate defects (cleft lip/palate), dental agenesis, ataxia, nystagmus, ear anomalies, hearing loss, visual defects, abnormal ocular movements
*FGF8/ FGFR1*	#612702 #147950	Cleft lip/palate, bone anomalies (syndactilia), dental agenesis
		Less frequently: hearing loss, bimanual synkinesia, ear anomalies, midline facial defects, choanal atresia, cardiac defects, coloboma
*PROK2/ PROKR2*	#610628 #244200	Sleep disorder, high-arched palate, bimanual synkinesia, hearing loss, pectus excavatum, hypodontia, obesity, nystagmus
*CHD7*	#612370	Coloboma, heart defects, choanal atresia, retardation of growth, genital anomalies, and ear abnormalities
*NELF*	#614838	None
*HS6ST1*	#614880	Cleft lip/palate, clinodactyly
*WDR11*	#614858	No
*SEMA3A*	#614897	No

*OMIM, Online Mendelian Inheritance in Man locus, gene and phenotype numbers (http://www.ncbi.nlm.nih.gov/omim)*.

Normosmic isolated hypogonadotropic hypogonadism is the consequence of defects in genes involved in the regulation and function of the GnRH neuron or the gonadotrope. Impaired GnRH production may result from mutations in the *GNRH1* gene or from defective regulation of the GnRH neuron by kisspeptin, neurokinin, or leptin signaling via their respective receptors. Mutations in the *GNRHR* gene, encoding the GnRH receptor present in the gonadotrope, are responsible for an impaired pituitary response to GnRH. In all the cases, except for defects in the neurokinin system, the secretion of both LH and FSH is impaired.

#### Cell-specific dysfunction: dissociated hypogonadism

##### Isolated LH deficiency

Congenital isolated LH deficiency with normal or high FSH production results from mutations in the *LHB* gene encoding the β subunit of LH (62, 63), and from defects in the neurokinin system responsible for the regulation of GnRH pulses. Neurokinin is a neuropeptide encoded by *TAC3*, which signals via the neurokinin receptor encoded by *TACR3* (64, 65). Micropenis and cryptorchidism may be observed, as a consequence of the fetal hypoandrogenism during the second and third trimesters, but there is normal testes volume in the newborn and child, because FSH levels are adequate. A mild form of isolated LH deficiency is the underlying pathophysiology of the “fertile eunuch” syndrome, characterized by the absence of signs of hypoandrogenism until puberty, when eunuchoid proportions become apparent in males with normal testis volume and sperm production. Mutations in *GNRHR* (66) and *LHB* (67) genes have been described.

##### Isolated FSH deficiency

Male fetuses with insufficient FSH may develop small testes during the second and third trimesters owing to Sertoli cell hypoplasia. External genitalia do not show signs of hypoandrogenism since LH production is normal or elevated (68).

### Combined or dual (primary and central) hypogonadism

DAX1 is a transcription factor encoded by *NR0B1* mapping to the short arm of the X chromosome. It has essential functions at several levels of the pituitary–gonadal and adrenal axes. DAX1 mutations result in a disorder characterized by adrenal hypoplasia and combined hypogonadism (Table 1). Testicular Sertoli and Leydig cell function is primarily affected resulting in moderately low hormone production; however, since the hypothalamic–pituitary axis is also defective, the gonadotrope is unable to increase LH and FSH production, despite the absence of an effective negative feedback loop.

Prader–Willi syndrome is another form of combined central and primary hypogonadism. This condition results from the lack of the paternally inherited chromosome 15 region q11-q13; this can be due to deletions in the paternal chromosome, to maternal disomy of 15q11-q13, or to a defective imprinting that silences the paternal chromosome 15. Several genes expressed exclusively from the paternal chromosome are believed to be involved in this syndrome (including *MAGEL2*, *MKRN3*, *NDN*, *SNURF-SNRPN*, and the *HBII* genes), although their underlying mechanism is not well understood (69). Hypogonadism is reflected in signs such as micropenis, cryptorchidism, scrotal hypoplasia, and microorchidism (70). However, the pathophysiology seems to be heterogeneous, and hypogonadism may be observed earlier or later in life, with a diverse participation of the hypothalamic–pituitary axis (19, 71–74).

## Diagnostic Assessment of Fetal-Onset Male Hypogonadism

The clinical and laboratory assessment of boys with suspected hypogonadism shows a wide spectrum and varies according to the etiology of the condition. Signs of hypoandrogenism are common to all; however, as already discussed, these signs will vary according to the period of fetal life in which hypogonadism is established. On the other hand, the evaluation of the other testicular hormones, and of any associated non-reproductive phenotype, may be extremely helpful in the diagnostic assessment of these boys.

### Patients with ambiguous or undervirilized external genitalia

If fetal hypogonadism is the underlying cause for the existence of a DSD presenting with ambiguous or insufficiently virilized genitalia (i.e., hypospadias, bifid scrotum), the condition can only be due to primary gonadal failure. The need for a differential diagnosis between testicular dysgenesis (i.e., whole gonadal dysfunction) and a specific steroidogenic failure emerges. A few clinical signs can be helpful in certain cases: the existence of two palpable gonads >1 mL is highly indicative of non-dysgenetic DSD (75), whereas the association of syndromic phenotypes – like skeletal dysplasia, macro/microcephaly, cardiac or renal defects, thalassemia, mental retardation, or minifascicular neuropathy orientate to gonadal dysgenesis (Table 2). Skeletal dysmorphisms may be present in patients with POR deficiency associated with the Antley–Bixler syndrome. Association with adrenal insufficiency is indicative of a non-dysgenetic steroidogenic defect (StAR, P450scc, P450c17, POR, 3β-HSD), although mutations in SF1 resulting in gonadal dysgenesis are also a possible cause.

**Figure 4:**
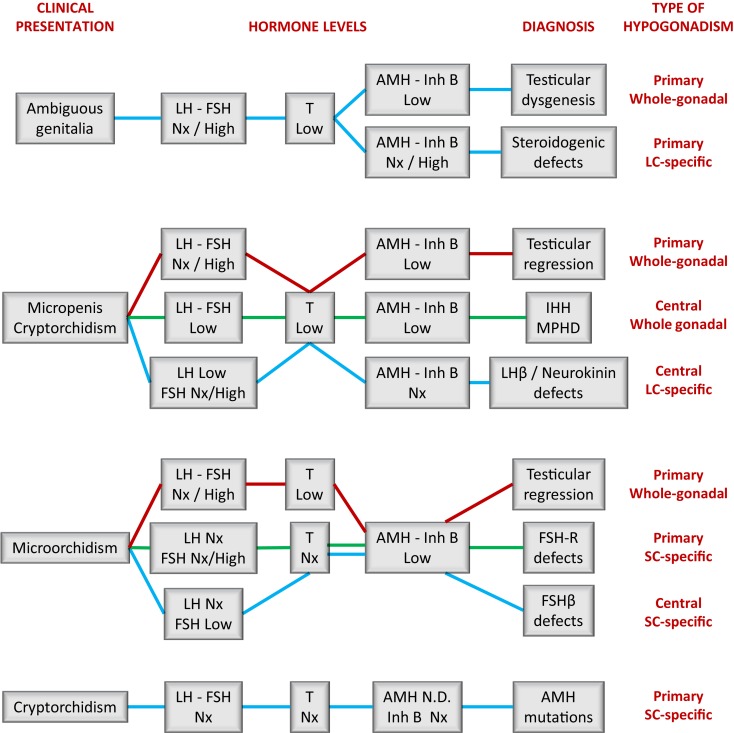
**A schematic guide for the interpretation of serum hormone levels in patients with fetal-onset male hypogonadism**. IHH, isolated hypogonadotropic hypogonadism; Inh B, inhibin B; MPHD, multiple pituitary hormone deficiency; Nx, normal. Levels are considered as high, normal, or low as compared to the reference levels for newborns or prepubertal boys.

Results from hormonal laboratory assessment in the newborn and infant should be interpreted according to reference values for age. In DSD patients, this is particularly relevant in the first month of life (Figure 2) (15), when patients are studied for diagnosis. During the first 3–6 months after birth, basal hormone level determinations may be helpful (Figure 4). The existence of normal levels of testosterone, AMH, and inhibin B rule out testicular dysfunction, and other etiologies of DSD should be sought (76, 77). When all testicular hormones are low and gonadotropins are elevated, gonadal dysgenesis is most likely [reviewed in Ref. (77)]. Low testosterone (53) with normal or elevated AMH (78) is characteristic of Leydig cell-specific hypogonadism. A prolonged hCG test (six IM injections every other day) and an ACTH test are necessary to distinguish between LHCG-R, StAR, and steroidogenic enzyme defects (Table 3). Gonadotropin levels may be somewhat elevated in the first months of life but they are usually normal during childhood in patients with steroidogenic defects (53). This is another example where primary hypogonadism is not hypergonadotropic in pediatric patients.

### Patients with male genitalia

The existence of normal male external genitalia rules out a fetal primary hypogonadism established in the first trimester, except for the rare form of Sertoli cell dysfunction due to *AMH* mutations leading to PMDS (54). PMDS patients were most frequently present with bilateral cryptorchidism; serum AMH is undetectable but the other reproductive hormones are within the normal range for age.

Fetal-onset central hypogonadism and primary hypogonadism established in the second or third trimester have clinical signs of hypoandrogenism as a common feature: small penis and undescended gonads. Microorchidism can be indicative of insufficient FSH stimulus – i.e., central hypogonadism – or of a testicular regression syndrome – i.e., a primary hypogonadism that can progress to anorchism. Because the hypothalamic–pituitary–testicular axis remains active for 3–6 months after birth (17, 18), this period represents a window of opportunity to establish the diagnosis of hypogonadism (1). However, the diagnosis can still be suspected and confirmed during the rest of infancy and childhood.

In some cases, the clinical presentation with cholestasis and/or hypoglycemia in the newborn or failure to thrive in infants can orientate the diagnosis to multiple pituitary hormone deficiency. Associated malformations in cerebral and hypothalamic-pituitary regions found on magnetic resonance imaging can be of further help (Table 4). A familial history of anosmia/hyposmia is suggestive of the diagnosis of isolated central hypogonadism, which could be reinforced by some anatomical or neurodevelopmental features in the infant or child (Table 5). Associated primary adrenal failure could orientate to adrenal hypoplasia congenital due to DAX1 mutations, whereas neonatal hypotonia and developmental delay may be indicative of Prader–Willi syndrome.

#### In childhood, primary hypogonadism does not equate to hypergonadotropic hypogonadism

The endocrine laboratory is necessary to certify the diagnosis of male hypogonadism. Basal gonadotropins, testosterone, and INSL3 are useful until the age of 3–6 months; thereafter dynamic stimulation tests are necessary to assess them. On the contrary, the Sertoli cell markers, AMH and inhibin B, are informative all through infancy and childhood without the need for stimulation tests. As discussed earlier, the occurrence of micropenis and non-palpable gonads prompts the differential diagnosis between central hypogonadism and testicular regression after the first trimester (Figure 4). If the patient is <3–6 months old, low levels of gonadotropins and Leydig and Sertoli cell hormones are suggestive of central hypogonadism (16, 79, 80), whereas high gonadotropins associated with low/undetectable testicular hormones are diagnostic of primary hypogonadism. After the age of 6 months, basal testosterone and INSL3 are no longer informative because they are normally low/undetectable during the rest of infancy and childhood. Low gonadotropins also lose usefulness. Undetectable AMH (81–83) and inhibin B (83, 84) are diagnostic of anorchia. The elevated levels of LH and FSH observed in these boys during the first years of life can subsequently decline to normal levels; therefore, serum gonadotropins within the reference range for age may not be informative during childhood (29). This is another clear example in pediatrics where primary hypogonadism is not hypergonadotropic.

#### Central hypogonadism is not always hypogonadotropic

The presence of micropenis, cryptorchidism, and microorchidism should prompt an early diagnosis of central hypogonadism, from which two main benefits may derive: first is to orientate the diagnosis of multiple pituitary hormone deficiency, favoring the opportune hormone replacement treatment (thyroid hormone, hydrocortisone, growth hormone). Second, as it has been postulated that the neonatal gonadotropic surge is physiologically important for testicular activity later in puberty and adulthood (85), early treatment with recombinant FSH and LH or hCG could be beneficial (79, 80). This also applies to isolated central hypogonadism. Analogously to the usefulness of testosterone and INSL3 to monitor Leydig cell response to LH/hCG (16), AMH (86) and inhibin B (87) are excellent markers of Sertoli cell response to FSH. In patients with a suspicion of central hypogonadism, AMH and inhibin B levels are suggestive if low but do not rule out the diagnosis if normal (88).

The hypoandrogenic states leading to micropenis and cryptorchidism – resulting from isolated LH deficiency due to mutations in the LHβ subunit or in the neurokinin system – are characterized by low LH and testosterone, but normal or elevated FSH. Interestingly, this central form of hypogonadism can even be hypergonadotropic, as observed in a young patient with delayed puberty, who had a functionally inactive but immunoreactive LH resulting in elevated serum levels associated with low testosterone (89).

Conversely, congenital isolated FSHβ deficiency, which presents with microorchidism but normal penile size and scrotal testes, has undetectable FSH and low inhibin B in adults with normal androgen with high LH after puberty (89, 90). No reports exist in childhood.

## Concluding Remarks

Fetal hypogonadism of the first trimester is primary and results in dysgenetic or cell-specific forms of DSD. In the second and third trimesters, primary and central hypogonadism share signs of hypoandrogenism and defective INSL secretion – i.e., micropenis, hypoplastic scrotum and cryptorchidism – and of Sertoli cell hypoplasia – i.e., microorchidism. In prepubertal patients, classical serum markers, like gonadotropins and testosterone, are helpful essentially during the first 3–6 months of life. With the advent of AMH and inhibin B, a biochemical diagnosis can also be envisaged during the rest of childhood. Clinical findings may also help in the diagnosis beyond early infancy. Finally, the pediatrician should not expect elevated gonadotropin levels during childhood to foresee a primary hypogonadism.

## Author Contributions

All authors contributed to manuscript writing and approved the final version.

## Conflict of Interest Statement

Rodolfo A. Rey and Patricia Bedecarrás have received honoraria from CONICET (Argentina) for technology services using the AMH ELISA. All other authors report no conflicts of interest.
